# Vitamin B_12_ deficiency in diabetic patients treated with metformin: A narrative review

**DOI:** 10.1007/s11845-024-03634-4

**Published:** 2024-02-21

**Authors:** Mazhar Salim Al Zoubi, Rasha Al Kreasha, Sarah Aqel, Ahmad Saeed, Ahmad R. Al-Qudimat, Raed M. Al-Zoubi

**Affiliations:** 1 Department of Basic Medical Sciences, Faculty of Medicine, Yarmouk University, Irbid, 211-63 Jordan; 2 Department of Basic Medical Sciences, Faculty of Medicine, Yarmouk University, Irbid, 211-63 Jordan; 3 Surgical Research Section, Department of Surgery, Hamad Medical Corporation, 3050 Doha, Qatar; 4 Department of Biomedical Sciences, College of Health Sciences, QU-Health, Qatar University, Doha, 2713 Qatar; 5 Department of Chemistry, Jordan University of Science and Technology, P.O.Box 3030, Irbid, 22110 Jordan

**Keywords:** B12 deficiency, Diabetes mellitus, Metformin, T2DM

## Abstract

Metformin is the most prescribed oral hypoglycemic drug and is considered by many health practitioners as the first-line treatment for non-insulin-dependent diabetes mellitus (T2DM). It is used either as a monotherapy or adjuvant to other anti-hyperglycemic agents. Most of its side effects are usually mild and self-limiting. However, several studies have shown an association between the use of metformin and low vitamin B_12_ levels in diabetic patients. The current review aimed to provide a literature review of the current published reports on the association, the possible mechanisms, and the related individualized risk factors that might lead to this incidence. The most accepted mechanism of the effect of metformin on vitamin B_12_ level is related to the absorption process where metformin antagonism of the calcium cation and interference with the calcium-dependent IF-vitamin B_12_ complex binding to the ileal cubilin receptor. In addition, many risk factors have been associated with the impact of metformin on vitamin B_12_ levels in diabetic patients such as dose and duration where longer durations showed a greater prevalence of developing vitamin B_12_ deficiency. Male patients showed lower levels of vitamin B_12_ compared to females. Black race showed a lower prevalence of vitamin B_12_ deficiency in metformin-treated patients. Moreover, chronic diseases including T2DM, hyperlipidemia, coronary artery disease, polycystic ovary disease (PCOD), obesity, and metformin therapy were significantly associated with increased risk of vitamin B_12_ deficiency.

**Graphical abstract:**

Metformin impacts vitamin B_12_ by (A) inhibiting calcium-dependent IF-B12 binding. (B) Prolonged use raises deficiency risk. (C) Males have lower B12 levels than females. (D) Black individuals show lower deficiency rates. (E) Conditions like T2DM, hyperlipidemia, coronary artery disease, PCOD, obesity, and metformin use heighten deficiency risk.

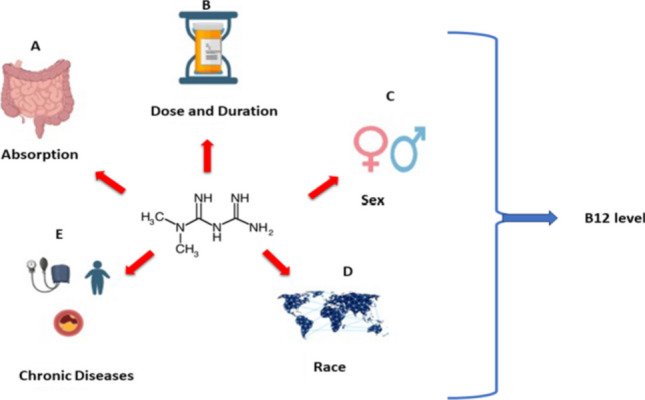

## Introduction

Diabetes mellitus is a complex metabolic disorder. The major clinical manifestation is chronic hyperglycemia which results from impaired insulin secretion or/and impaired insulin action. Diabetes mellitus classifications include type 1 insulin-dependent, type 2 insulin-independent, gestational diabetes, and other less common types (e.g., MODY) [1]. In addition, certain criteria for the diagnosis of diabetes mellitus are shown in Table 1 [2]. Generally, type 2 diabetes mellitus (T2DM) is a global health concern that is steadily rising [3]. For instance, an estimated 422 million adults with diabetes worldwide was reported in 2014 [4]. Diabetes prevalence expanded from 4.7% in 1980 to 8.5% in 2014 in adults, with the greatest increase in low and middle-income countries compared to high-income nations [5]. Additionally, 1.1 million children and adolescents aged 14–19 years have T1DM, as estimated by the International Diabetes Federation (IDF), and without interventions to stop the rise in diabetes, by 2045, there will be at least 629 million diabetic patients [6]. Type 2 diabetes mellitus is one of the leading causes of morbidity and mortality worldwide, and it is associated with many systemic vascular complications, which can reduce the quality of life and result in social and economic burdens [7–10]. Moreover, the financial cost of the healthcare of diabetes mellitus is another economic burden. For. Instance, in many countries, around 5–10% of the healthcare budget is assigned for the treatment of diabetes mellitus [11].

**Table 1:** Summary of metformin uses and precautions

**Recommendations**	**Precautions**	**Contraindications**
**Type 2 DM in adults and children ≥ 10 years**	Serum creatinine > 1.5 mg/dL for men and > 1.4 mg/dL for women	eGFR < 30 mL/min/1.73 m^2^
**Prediabetes ± BMI ≥ 35 kg per m**^**2**^	Vitamin B_12_ deficiency	
**Gestational diabetes**	Intravenous contrast administration [12]	

Vitamin B_12_ deficiency is a significant concern in diabetic patients, particularly those treated with metformin. Several studies have highlighted the association between metformin use and vitamin B_12_ deficiency in individuals with type 2 diabetes mellitus [13–22]. The prevalence of vitamin B_12_ deficiency in diabetic patients on metformin has been reported to be as high as 93% [13]. Furthermore, the impact of vitamin B_12_ deficiency on peripheral neuropathy in diabetic patients has been a subject of investigation, with studies demonstrating an association between vitamin B_12_ deficiency and peripheral neuropathy in individuals with type 2 diabetes mellitus [14, 18, 19, 23]. Additionally, the prevalence of vitamin B_12_ deficiency has been found to be higher in diabetic patients compared to the general population [24]. This deficiency has also been linked to gastroparesis in patients with type 2 diabetes [25]. Moreover, the prevalence of vitamin B_12_ deficiency has been reported to be higher in individuals with pre-diabetes and diabetes compared to those without these conditions [26]. These findings underscore the importance of routine screening for vitamin B_12_ deficiency and the potential need for supplementation among diabetic patients, especially those on metformin therapy [27, 28]. In recent years, there has been increasing interest in the association between metformin use and vitamin B_12_ deficiency in patients with type 2 diabetes mellitus (T2DM)[15, 18]. Several studies have investigated the prevalence of vitamin B_12_ deficiency and its associated factors among patients with T2DM who are on metformin [20, 29–31]. Some studies have indicated a correlation between longer duration of metformin use and increased risk of vitamin B_12_ deficiency [15, 32, 33]. Additionally, there is evidence suggesting a link between metformin use and diabetic neuropathy [18, 19, 21, 30]. The American Diabetes Association guidelines now recommend routine evaluation for vitamin B_12_ deficiency in patients taking metformin [19, 29]. It has been suggested that physicians should consider screening for vitamin B_12_ deficiency in diabetic patients before starting metformin therapy, and periodic monitoring of vitamin B_12_ levels has been recommended for all patients using metformin, particularly for those using the medication long-term [21, 34]. Moreover, the potential role of vitamin B_12_ deficiency in exacerbating conditions such as diabetic neuropathy and gastroparesis in patients with T2DM has been highlighted, emphasizing the importance of addressing this issue in clinical management [25]. The research on vitamin B_12_ deficiency in diabetic patients treated with metformin underscores the need for increased awareness and monitoring of vitamin B_12_ levels in this patient population. The evidence suggests a potential association between metformin use and vitamin B_12_ deficiency, with implications for the management of diabetic patients. Hence, this review provides additional insights into the mechanisms underlying this association and guides the development of targeted interventions to mitigate the risk of vitamin B_12_ deficiency in diabetic patients using metformin.

## Methods

We searched Google Scholar and PubMed using the keywords metformin, diabetes, vitamin B_12_, deficiency, metabolism, mechanism, risk factors, and side effects. We excluded articles that were not related to our research and the ones that did not have sufficient data. In the end, 25 articles were included from the years 2009 to 2021. Out of the 25 articles, 21 showed an association between the use of metformin and vitamin B_12_ deficiency, while 4 did not. All clinical trials included the use of metformin. However, not always the participants were diabetics. Vitamin B_12_ deficiency percentage was calculated in some of the studies and others were not. Table 2 summarizes the main data.

**Table 2:** Summary of literature that researched the association of vitamin B_12_ with metformin use

**Study**	**Result**	**Vit B**_**12**_ **deficiency**	**Population size**	**On metformin**	**Diabetic no metformin**	**Non-diabetic**
**Soutelo et al.** [35]	Associated	-	296	178	-	118
**Ali et al.** [36]	Associated	10.71%	280	140	-	140
**Sakyi et al.** [37]	Associated	40.5%	200	200	-	-
**Miyan and Waris** [38]	Associated	3.9%	932	645	287	-
**Lata Kanyal et al.** [39]	Associated	-	100	45	-	55
**Kim et al.** [40]	Associated	22.2%	1111	1111	-	-
**Alvarez et al.** [19]	Associated	7.3%	162	162	-	-
**Zalaket et al.** [41]	Associated	22.5%	200	200	-	-
**Alharbi et al.** [18]	Associated	9.4%	412	319	93	-
**Khan et al.** [42]	Associated	29.66%	209	209	-	-
**Ahmed et al.** [14]	Associated	28.1%	121	121	-	-
**Beulens et al.** [43]	Associated	28.1%	550	550	-	-
**Ko et al.** [44]	Associated	9.5%	799	799	-	-
**Sato et al.** [45]	Associated	13%	84	46	38	-
**de Groot-Kamphuis et al.** [46]	Associated	14.1%	298	164	-	134
**Romero and Lozano** [47]	Associated	8.6%	109	81	-	28
**Reinstatler et al.** [48]	Associated	5.8%	8488	575	1046	6867
**Liu et al.** [49]	Associated	29%	134	56	-	78
**Kancherla et al.** [50]	Associated	7%	26,115	3687	-	-
**Wile et al.** [51]	Associated	31%	122	59	-	63
**de Jager et al.** [52]	Associated	19%	256	127	129	194
**Rodríguez-Gutiérrez et al.** [53]	Not associated	2%	150	50	-	-
**Raizada et al.** [54]	Not associated	-	183	121	-	63
**Adetunji et al.** [55]	Not associated	-	520	279	241	-
**Elhadd et al.** [56]	Not associated	8%	362	235	64	

## Result

### Metformin

**Fig. 1:**
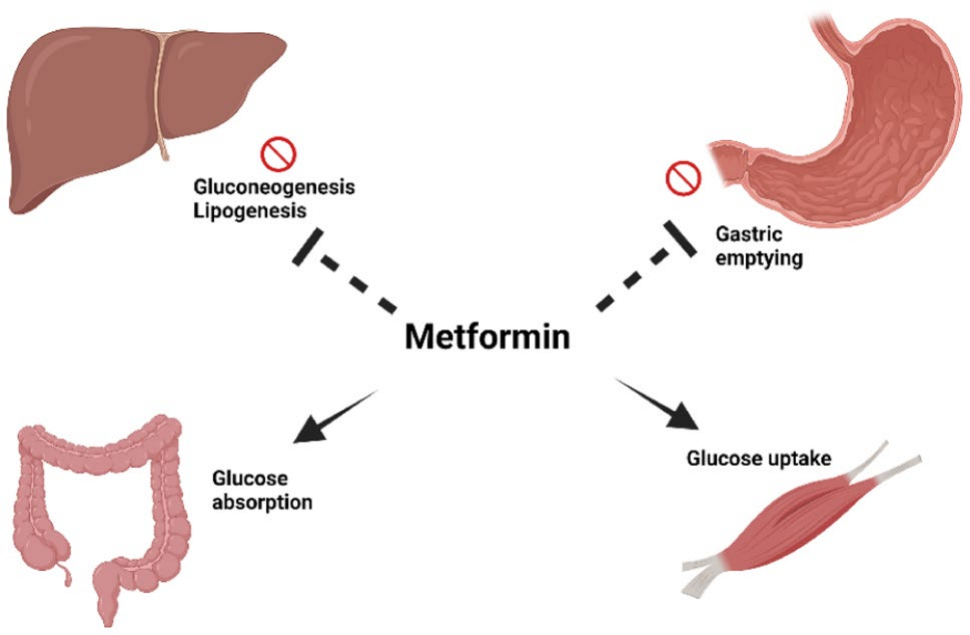
Illustration of metformin’s mechanism of action

Metformin belongs to a group of oral hypoglycemic drugs called biguanides. Some aspects of metformin’s mechanism of action are still not fully understood [57]. It is suggested that metformin primarily achieves its glucose-lowering effects through the inhibition of gluconeogenesis in the liver, therefore lowering the production of glucose. Other mechanisms include increased insulin sensitivity [58] and inhibiting lipogenesis [59] as well as increasing the uptake of glucose in the intestine [60] and muscles. In addition, metformin delays gastric emptying which contributes to a decrease in appetite [61]. Figure 1 illustrates metformin’s mechanism of action. A summary of metformin’s mechanism of action is shown in Fig. 1.

Metformin is routinely prescribed to 120 million patients around the world, as it is the first-line treatment for individuals with diabetes mellitus (T2DM) and normal kidney function [62, 63]. It is currently the most prescribed oral anti-diabetic agent and recommended as first-line therapy for type 2 diabetes because of its safety, effectiveness, and possibility for use in combination with other anti-diabetic medications [23]. Both the American Diabetes Association and the European Association for the Study of Diabetes recommend the use of metformin as the first therapeutic choice in the management of type 2 diabetes mellitus (T2DM). Metformin enhances peripheral insulin sensitivity and cardiovascular mortality risk and contributes to weight loss. Medical situations that may require the prescription of metformin, as well as the precautions of its use, are shown in Table 2. The majority of the side effects associated with metformin are mild. One or more episodes of nausea, vomiting, or diarrhea are experienced in 30–45% of patients [64]. Other less common side effects include headache, diaphoresis, weakness, and rhinitis[58].

### Vitamin B_12_

Vitamin B_12_ also called cobalamin is the largest and most complex vitamin known. It is a water-soluble vitamin with a molecular weight of 1355.4 [65, 66]. It is found mainly in animal sources such as meat, milk, egg, fish, and shellfish, explaining why strict vegetarians are highly prone to developing vitamin B_12_ deficiency [67]. However, it can also be found in large quantities in some plants like edible algae or blue-green algae [68].

**Fig. 2:**
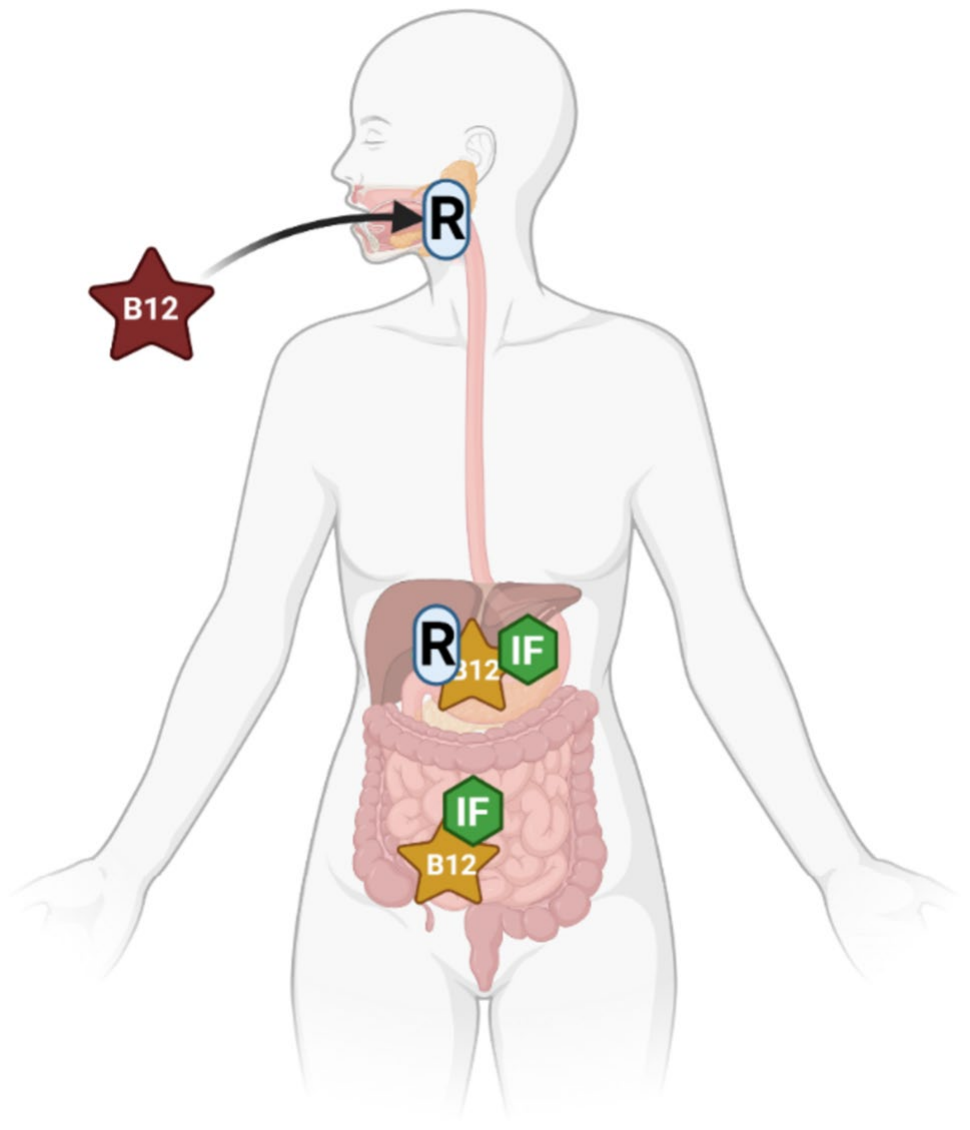
Illustration of absorption pathway of vitamin B_12_ in the human body

Cobalamin is introduced to the body through the oral cavity, where it binds to its first carrier protein known as transcobalamin I or R-protein. R-protein protects cobalamin from gastric acidity and low pH in the stomach. Gastric parietal cells secret the intrinsic factor IF, the second carrier of cobalamin. In the small intestine, the R-protein is hydrolyzed releasing cobalamin which then binds to intrinsic factors. The cobalamin-IF complex is then absorbed in the distal ileum [65, 69]. An illustration of vitamin B_12_ metabolism is shown in Fig. 2. There are many ways to diagnose vitamin B_12_ deficiency such as identification of macrocytic anemia and findings of hyper-segmented (more than 5 lobes) neutrophils on a blood smear. Nevertheless, the most sensitive and diagnostic criteria are low levels of serum cobalamin (< 148 pmol/L)[70]. Mild vitamin B_12_ deficiency can result in fatigue, weakness, and memory loss. Severe deficiency can result in macrocytic anemia, peripheral neuropathy, and mental psychiatric changes [71–73]. The prevalence of deficiency varies by age group. In the US, 3% of those aged 20–39, 4% of those aged 40–59, and 6% of those aged 70 have vitamin B_12_ deficiency [74]. On the other hand, it is estimated that the subclinical deficiency of vitamin B_12_ in the US ranges from 10–15% among those > 60 years old to 23–35% in > 80 years old [75]. In Asian and African countries, the prevalence is much higher; for example, in India, 70% of adults are deficient [74].

The deficiency of vitamin B_12_ can arise from its lack in one’s diet or a defect in gastrointestinal absorption [76]. Some genetic studies reported an association between certain genetic variants and the deficiency of vitamin B_12_ [77, 78]. Other risk factors that have been suggested to be related to the development of vitamin B_12_ deficiency include pernicious anemia and the long-term use of certain drugs such as acid-suppressing medications [79] and metformin [52].

### Metformin and vitamin B_12_ deficiency

Metformin-induced vitamin B_12_ deficiency was reported as early as 1971 when Tomkin et al. recommended that all patients on long-term metformin therapy should be tested for serum B12 deficiency annually [80]. In a randomized placebo-controlled trial, metformin treatment was associated with a mean decrease in vitamin B_12_ concentration by 19% and an increase in homocysteine concentration by 5% [52]. Former studies have reported that the prevalence of vitamin B_12_ deficiency among metformin-treated patients varied greatly and ranged between 5.8% and 52% [43, 46–49, 52, 81–83]. A recent meta-analysis that included thirty-one studies reported that patients who received metformin had a significantly higher risk of vitamin B_12_ deficiency in comparison with diabetic patients not taking metformin, and significantly lower serum vitamin B_12_ concentrations which depended on dose and duration of treatment [84]. In a retrospective study, it was observed that subjects receiving doses of metformin higher than 2000 mg/day or for more than 4 years had low levels of vitamin B_12_ [18]. In another review of patients with type 2 diabetes taking metformin after up to 4 months, they showed a decrease in B_12_ level by 57 pmol/L, which would be predicted to lead to a frank deficiency in a significant proportion of patients based on European data for B_12_ status [85].

Contrary to the increase in homocysteine levels that is stated above, a cross-sectional study that concluded 1111 patients with type 2 diabetes who took metformin for at least 6 months reported that homocysteine levels were negatively correlated with vitamin B_12_ levels, and suggested a hypothesis that B_12_ deficiency due to the use of metformin occurred at the tissue level [40]. While numerous studies reported an association between vitamin B_12_ deficiency and metformin treatment, in 2017, Rodríguez-Gutiérrez et al., found no variation in vitamin B_12_ levels between participants receiving metformin and those naive to therapy [53].

## Suggested mechanisms

### Absorption

Different mechanisms have been suggested clarifying how metformin interposes with vitamin B_12_ absorption. In 1977, Caspary and Creutzfeldt proposed a mechanism that was how bacterial overgrowth in the intestine resulted in bacterial binding with IF-B12 complex instead of the latter getting absorbed [86]. Another mechanism that was suggested is the alteration of metformin on intestinal motility, thereby reducing the absorption of vitamin B_12_ [87]. The process of B_12_-intrinsic factor complex uptake is known to be dependent on calcium availability. Therefore, out of the many mechanisms that were suggested of how metformin interferes with the absorption of vitamin B_12_, metformin antagonism of the calcium cation and interference with the calcium-dependent IF-vitamin B_12_ complex binding to the ileal cubilin receptor was the most accepted one. Bauman et al. suggested that the protonated metformin molecule directs itself towards the hydrocarbon core of the ileal cell membrane, displacing the divalent calcium cations by giving a positive charge to the membrane surface. An effect that can be reversed by increasing calcium intake, consequently greatly supporting the mechanism [88].

### Dose and duration

Many studies stated that serum vitamin B_12_ concentrations are inversely related to long-term therapy and/or higher doses of metformin use [50, 52, 80, 89]. In addition, a meta-analysis of four clinical trials demonstrated that after three to 6 months of metformin use it significantly reduced vitamin B_12_ levels [85]. Several studies found an association between metformin dose and B_12_ deficiency, while there was no correlation with its duration. Higher doses of metformin were associated with lower levels of vitamin B_12_. Hence, it is important to consider metformin dose in recommendations for screening for cobalamin deficiency [19, 35, 40, 43]. Nevertheless, other research demonstrated a relationship between metformin usage, with higher doses, and longer durations, showing a greater prevalence of developing vitamin B_12_ deficiency [18, 41, 84].

### Other risk factors

Alvarez et al. found that male patients had lower levels of vitamin B_12_ in comparison to females [19]. Black race was found to be a protective factor for vitamin B_12_ deficiency in metformin-treated patients [14]. A meta-analysis by Niafar et al. found that patients with T2DM, hyperlipidemia, coronary artery disease, polycystic ovary disease (PCOD), or obesity, and on metformin therapy were significantly associated with increased risk of vitamin B_12_ deficiency and lower serum vitamin B_12_ concentrations [9]. Increased metformin exposure was hypothesized to be associated with lower levels of B12 and more severe peripheral neuropathy [51]. Impaired vibration sensation and proprioception and paresthesia are unfortunately similar in both diabetic neuropathy and vitamin B_12_ deficiency [71]. Consequently, it has been suggested that serum B12 levels should be screened routinely in long-term metformin users [82].

In a comparison between T2DM patients having neuropathy, and those who do not, it was found that the first group had higher levels of B_12_ deficiency than the other group [35]. Moreover, in a dose-dependent manner, both borderline and low levels of vitamin B_12_ occurred to be associated with the presence of distinct neuropathies and macrocytic anemia [41]. T2DM patients with neuropathy treated with MET 1000 mg/d manifested lower levels of vitamin B_12_ [35]. Diabetic neuropathy relationship with vitamin B_12_ deficiency has prominent importance considering vitamin B_12_ deficiency is profoundly common, especially among patients with diabetic neuropathy. Furthermore, diabetic or pre-diabetic patients diagnosed with diabetic neuropathy may have neuropathy due to vitamin B_12_ deficiency. Therefore, before initiating the treatment of diabetic neuropathy, the other condition should be excluded [19]. A cross-sectional study stated that patients possibly get diagnosed with diabetic neuropathy instead of vitamin B_12_ deficiency induced by metformin which leads to neurologic damage with symptoms of peripheral neuropathy [39].

On the other hand, a cross-sectional study by Ahmed et al. found that there was no difference among those with normal and decreased vitamin B_12_ levels and the presence of neuropathy [14]. Smoking was reported to be associated with lower vitamin B_12_ levels than in non-smokers (Table 3) [42].

**Table 3:** Smoking and low vitamin B_12_ levels in metformin-treated patients

**Study**	**Metformin users and vitamin B**_**12**_ **deficient (*n*)**	**Smokers (%)**
**Kim et al. **[40]	247	20.7%
**Khan et al. **[42]	62	62.90%
**Beulens et al. **[43]	126	16.7%
**Ko et al. **[44]	76	13.2%

### Cognitive impairment

A meta-analysis reported that cognitive impairment prevalence happened to be less significant in people with diabetes treated with metformin. Additionally, six studies showed that dementia incidence also had a reduced risk. Campbell et al. reported that there is no available evidence supporting the use of metformin by non-diabetic individuals in an attempt to prevent dementia. Nevertheless, in patients at risk of developing dementia or Alzheimer’s disease, metformin should continue to be used as first-line therapy for diabetes [90]. A contradicted study, by Moore et al., reported a significant finding of impaired cognitive performance in diabetic patients treated with metformin, which might be alleviated by vitamin B_12_ and calcium supplements [91].

### Multivitamins

Individuals who are receiving supplementation of multivitamins may potentially have protection against B_12_ deficiency in comparison to those not receiving any [40, 42].

### PPIs and/or H_2_RAs

In consideration of the expanding prevalence of obesity, T2DM, and GORD, there is now more potential for the use of acid-suppressing medications and anti-diabetics concomitantly. Considering that the solitary use of either metformin, PPIs, or H_2_RAs, has been shown to considerably deplete vitamin B_12_, co-prescription of metformin with either PPIs or H2RAs can have additional adverse effects on vitamin B_12_ status [92]. The production of stomach acid by the gastric parietal cells is needed for the conversion of pepsinogen to pepsin, which releases vitamin B_12_ from ingested proteins. PPIs and H2RAs inhibit this acid production. PPIs block gastric H+K+-ATPase, which is responsible for pumping H+ ions from within gastric parietal cells into the gastric lumen, where they interact with Cl− ions to form HCl. On the other hand, H2RAs inhibit the interaction of histamine with the parietal cell histamine H2 receptor. This blocks the cAMP-dependent pathway that promotes H+K+-ATPase function, thus reducing gastric acid production. A reduction or lack of gastric acid and pepsin diminishes the release of vitamin B_12_ from food and hence decreases its availability for absorption in the ileum [93]. Long et al. observed that the association of vitamin B_12_ deficiency along with the concomitant use of metformin and proton pump inhibitors was significantly greater than those on monotherapy. 34.15% of patients with co-prescription were vitamin B_12_ deficient; in contrast, those on metformin (21.91%) or PPIs (25.58%) monotherapy had lesser deficiency suggesting an additional impact [94]. Nevertheless, there is no clear indication that biochemical or functional vitamin B_12_ deficiency would occur due to decreased serum vitamin B_12_ that is caused by these medications, as indicated by circulating homocysteine and methylmalonic acid concentrations, or to the hematologic and neurological manifestations of clinical deficiency [93]. Until other studies are done, Miller recommends those who are co-prescribed to these drugs to observe vitamin B_12_ status and take vitamin B_12_ supplements if needed [93]. However, Romero and Lozano found that there was no notable variation in plasma vitamin B_12_ levels among those receiving and not receiving PPIs [47].

### Sulfonylurea and/or insulin

In a cross-sectional study by Kang et al., it was demonstrated that T2DM patients need to monitor their vitamin B_12_ deficiency and keep an ordinary regulation of their vitamin B_12_ levels especially those who were prescribed metformin in combination with sulfonylurea. in contrast to insulin metformin and sulfonylurea, co-prescription has been shown to decrease the mean blood vitamin B_12_ level and the prevalence of vitamin B_12_ deficiency was significantly increased. Moreover, even after modifications for the daily dosage and duration of metformin among the patients taking the maximal dosage of sulfonylurea, this finding persisted to be significant [83].

### Rosiglitazone

In a 6-week study to find the impacts of treatment with metformin or rosiglitazone on serum concentrations of homocysteine, folate, and vitamin B_12_ in patients with recently diagnosed T2DM where 165 patients have been tested, Sahin et al. observed that metformin use was associated with an increase in homocysteine levels, but vitamin B_12_ did not vary significantly. Whereas management with rosiglitazone showed a decrease in homocysteine levels, with no significant change in vitamin B_1_ levels [95].

## Conclusion

Until this day, some of the aspects of metformin’s mechanism of action are still not fully understood*.* We found in our review that there is an undeniable association between the use of metformin and the progression of vitamin B_12_ deficiency in some diabetic patients. However, the benefits outweigh the risks. When spotted early, vitamin B_12_ deficiency is easily treated. We recommend further studies to achieve a better understanding of the possible mechanisms, risk factors, and relation of vitamin B_12_ deficiency to the dose and duration of metformin use. In addition, we advise physicians and health practitioners to always be aware of these side effects. Routine monitoring of vitamin B_12_ for patients on long-term metformin was also suggested by other studies. These recommendations were associated with higher dosages and longer durations of usage. Some of these studies recommended the regular check for doses ranging more than 1000 to 2000 mg/day, while others did not specify a certain dose or duration. Screening was mostly advised to be annual [14, 18, 19, 36, 39, 41, 43–45, 49, 52, 83, 93, 96]. On the contrary, a recommendation for screening by Rodríguez-Gutiérrez et al. could not be made [53].

## Data Availability

The data that supports the findings in this study are available from the corresponding authors upon reasonable request.
